# A Rational Design of a Biphasic Dissolution Setup—Modelling of Biorelevant Kinetics for a Ritonavir Hot-Melt Extruded Amorphous Solid Dispersion

**DOI:** 10.3390/pharmaceutics12030237

**Published:** 2020-03-06

**Authors:** Alexander Denninger, Ulrich Westedt, Jörg Rosenberg, Karl G. Wagner

**Affiliations:** 1 Department of Pharmaceutical Technology and Biopharmaceutics, University of Bonn, Gerhard-Domagk-Strasse 3, 53121 Bonn, Germany; 2 AbbVie Deutschland GmbH & Co. KG, Knollstrasse 50, D-67061 Ludwigshafen, Germany

**Keywords:** biphasic dissolution, liquid-liquid-phase-separation, kinetic modelling, biorelevant media, hot melt extruded amorphous solid dispersion

## Abstract

Biphasic dissolution systems achieved good predictability for the in vivo performance of several formulations of poorly water-soluble drugs by characterizing dissolution, precipitation, re-dissolution, and absorption. To achieve a high degree of predictive performance, acceptor media, aqueous phase composition, and the apparatus type have to be carefully selected. Hence, a combination of 1-decanol and an optimized buffer system are proposed as a new, one-vessel biphasic dissolution method (BiPHa+). The BiPHa+ was developed to combine the advantages of the well-described biorelevance of the United States Pharmacopeia (USP) apparatus II coupled with USP apparatus IV and a small-scale, one-vessel method. The BiPHa+ was designed for automated medium addition and pH control of the aqueous phase. In combination with the diode array UV-spectrophotometer, the system was able to determine the aqueous and the organic medium simultaneously, even if scattering or overlapping of spectra occurred. At controlled hydrodynamic conditions, the relative absorption area, the ratio between the organic and aqueous phase, and the selected drug concentrations were identified to be the discriminating factors. The performance of a hot-melt extruded ritonavir-containing amorphous solid dispersion (ritonavir-ASD) was compared in fasted-state dissolution media leading to different dissolution-partitioning profiles depending on the content of bile salts. An advanced kinetic model for ASD-based well described all phenomena from dispersing of the ASD to the partitioning of the dissolved ritonavir into the organic phase.

## 1. Introduction

Dissolution tests are commonly used in order to characterize the performance of formulations with respect to their dissolution kinetics [1]. It is an important tool guiding formulation development and/or quality control [2]. The relevance of dissolution results strongly depend on the presence of sink conditions. Otherwise, dissolution kinetics are compromised by the solubility of the drug. This circumstance involves a great challenge for dissolution methods to characterize drug products with poorly water-soluble active pharmaceutical ingredients (API). By using standard methods, several modifications are applied to prevent this sink limitation [3]:

1. Adding surfactant;
2. Using large volumes or flow-through apparatus;
3. Cosolvents;
4. pH-adjustment.

All these methods reveal considerable disadvantages. Surfactants and cosolvents can influence the behavior of the formulation in the aqueous medium [4,5,6]. Large volumes of dissolution medium are inconvenient to handle and non-economical. In many cases, pH adjustment does not lead to a significantly enhanced solubility and might not reflect the relevant pH of the absorption site. All listed approaches have less biopharmaceutical significance and are of limited use in pharmaceutical development.

Many approaches were established to overcome these challenges such as absorption models (PermeaLoop, [7]) non-sink models (pH dilution method [8]), or artificial stomach-duodenum models [9]. Especially the biphasic dissolution methods seem to be suitable to provide superior prediction of bioperformance [3,10,11]. In this method, the formulation disintegrates and dissolves under non-sink condition in the aqueous medium. The aqueous phase, in turn, is covered with an organic phase creating an absorption-sink. Currently, most setups are working with a pH-shift simulating the gastrointestinal passage [12,13,14]. This setup is capable of simulating the behavior of these formulations more meaningfully [15], which includes dissolution, precipitation, and absorption. The simplified setup of this quasi-sink method is one major advantage compared to other absorption methods such as bicompartmental systems [15] or the TNO Gastro-Intestinal Model (TIM) model [16]. Generally, the goal of this model is to establish a setup that is predictive in terms of extent and kinetics of the dissolution for in vivo pharmacokinetics. The relationship between the absorption rate in vivo to the absorption rate in vitro can be expressed for the absorption step in the biphasic model as follows [17]:

(1) $$k_{partitioning}=P_{in\;vitro}\frac{A_{aqua}}{V_{aqua}}\;\approx k_{absorption}=P_{in\;vivo}\frac{A_{intestinte}}{V_{intestine}}$$

where *k_partitioning_* is the partitioning rate constant, *A_aqua_/V_aqua_* is the ratio between the interface area and the volume of the aqueous phase in vitro, *k_absorption_* is the absorption rate constant, and *A_intestine_/V_intestinea_* is the ratio between the interface area and the intestinal volume area. *P_in vitro_* and *P_in vivo_* represent the permeability rate through the aqueous organic interface of the in vitro assay and the gastrointestinal permeability. By assuming a first-order absorption model, *k_partitioning_* can be set to in vivo comparable values of *k_absorption_* by adjusting the ratio of phase interface (*A_aqua_*) and volume of the aqueous phase (*V_aqua_*) according to Mudie et al. [17].

The purpose of this work was a rationale development of a fully automated, small-scale, one-vessel biphasic dissolution method, which generates biorelevant results, for the fasted state using a hot-melt extruded ritonavir-containing amorphous solid dispersion formulation (ritonavir-ASD). Thus, the most relevant geometrical parameters have been identified [17], which are necessary for scaling down at controlled hydrodynamics [18,19]. Emphasis was put on the development of biorelevant aqueous media suitable to keep buffer capacity constant over the entire pH range. As it is mandatory to change the pH in situ during the test, further focus was to achieve the above mentioned similar osmolarity and buffer capacity at a minimum volume change. The in vivo relevance was demonstrated on the basis of literature data. In the second part of this work, we extended a previously published kinetic model [13] to describe the processes of ritonavir dissolution and partitioning during the biphasic dissolution experiment and improve the understanding of the resulting concentration–time curves.

## 2. Materials and Methods

### 2.1. Materials

Neat ritonavir and ritonavir-ASD were provided by AbbVie Germany, Ludwigshafen, Germany. The ritonavir-ASD was prepared as extrudate beads by hot melt extrusion on a co-rotating twin-screw extruder, and composed of ritonavir (15%), copovidone (74%), sorbitan monolaurate (10%), and colloidal silicon dioxide (1%). The purity of the ingredients was according to the compendial specifications (Ph.Eur./USP). The absence of API-related crystallinity in the ASD was confirmed by polarized light microscopy (Model DMLM, Leica Microsystems, Wetzlar, Germany). Tri-potassium phosphate (Alfa Aesar, Kandel, Germany), tri-potassium citrate (Carl Roth, Karlsruhe, Germany), and sodium hydroxide (VWR Chemicals, Darmstadt, Germany) were used for the buffer concentrate. Lecithin, 1-decanol, and sodium taurocholate were obtained from Alfa Aesar (Kandel, Germany). Glitter powder was purchased from Jofrika Cosmetics, Bergisch-Gladbach, Germany. The high performance liquid chromatography HPLC chemicals consisted of methanol (VWR Chemicals, Darmstadt, Germany), acetonitrile (VWR Chemicals, Darmstadt, Germany), and demineralized water (Merck Milli-Q, Darmstadt, Germany). Samples for HPLC were filtered through a 0.45 µm cellulose acetate syringe filter (VWR Chemicals, Darmstadt, Germany).

### 2.2. HPLC Method

A HPLC-method was established to verify the dissolved amount of ritonavir in comparison to UV-spectrophotometric determination. A Waters diode array detector with a reversed phase column (Phenomenex Inertial ODS-3 150 × 4.6 mm, 5 µm) was used at 35 °C. The mobile phase contained 40% methanol, 40% acetonitrile, and 20% demineralized water. The quantification was performed in triplicates. All samples from the aqueous phase were filtered immediately after withdrawing the samples through a 0.45 µm cellulose-acetate syringe filter. The filters were pre-saturated by 10 mL of the aqueous phase to exclude adsorption of ritonavir to the membrane. After filtration, 5 µL of the samples were directly injected. The measurement was performed at 240 nm. The retention time was 7.0 min.

### 2.3. Equilibrium Solubility

The equilibrium solubility was determined using the shaking flask method for both the aqueous and the organic medium. The aqueous medium was adjusted to pH 1.0 and 6.8. For FaSSIF-V2, sodium taurocholate and lecithin were added as mentioned above. Ritonavir was added in excess. The flasks were shaken at 37 °C ± 0.5 °C for three days. All samples were analyzed as described in HPLC method (Section 2.2).

### 2.4. pKa Determination of Ritonavir

All pKa values were determined UV-spectrophotometrically using a Sirius T3 apparatus. The pKa values were identified, where a maximum change of UV-spectra was detected between pH 1.0 to 13.0. The titration was performed at room temperature in a 0.15 M potassium chloride/methanol mixture (ratios of 5/5, 4/6, 3/7). About 1 mg of ritonavir was used for each titration [20].

### 2.5. Fully Automated Biphasic Dissolution Apparatus (BiPHa+)

#### 2.5.1. General Method Design

The dissolution method was designed considering the following scaling factors, which were intended to be essential to describe a biphasic dissolution method:

1. Normalized absorption area:

(2) $$A_{norm}=\frac{aborption\;area}{aqueous\;volume}$$

2. Volume ration:

(3) $$R_{V}=\frac{volume\;organic\;phase}{volume\;aqueous\;phase}$$

3. Concentration of API

**Figure 1:**
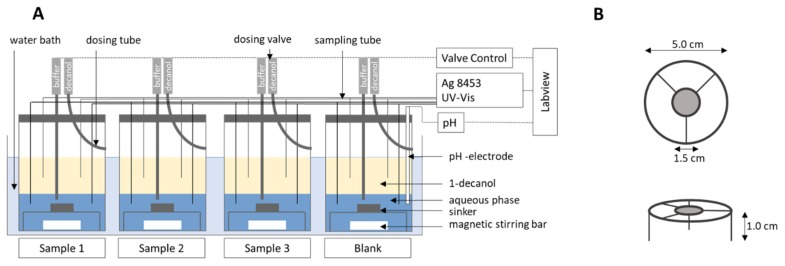
The BiPHa+ apparatus: (**A**) Biphasic dissolution setup; (**B**) dimensions of sinkers.

The BiPHa+ apparatus (Figure 1) contained four vessels: One blank and three sample vessels in a water bath at 37 °C ± 0.5 °C. Each vessel has a cylindrical shape with a diameter of 5.0 cm with a plain bottom. The filling height of both phases is 2.55 cm. The overall height of the cylindrical vessels is 10.0 cm. Due to its geometrical shape, the normalized interface area (*A_norm_*) can be adjusted by changing the aqueous volume (Equation (1)). An unstirred water layer within the aqueous phase must be avoided [19]. Therefore, adequate mixing was achieved by triangle magnetic stirrers, which enable a turbulent movement of the water in order to uniformly bathe the ritonavir-containing ASD sample. The stirring rate was set to 160 rpm, making sure no funnel was formed at the interface between the aqueous and organic phase. ASD samples were put in sinkers (mesh size 1 mm) directly above the stirrer (Figure 1). Adjusting the pH and covering 1-decanol was conducted fully automated by a liquid dispensing system (precision: ± 10 µL). Representative for the sample vessels, the pH-value during dissolution testing was controlled by a pH–electrode (Semi-mikro VWR Collection VWR Chemicals, Darmstadt, Germany) in the blank vessel. An Agilent 8454 diode array UV spectrophotometer (Agilent Technologies, Waldbronn, Germany) was used to quantify the drug substance in the aqueous and the organic layer. A LabView^®^ application was developed, which controls automated liquid dispensing including pH adjustment as well as UV-Vis detection and subsequent data processing of recorded spectra.

#### 2.5.2. Quantification and Scattering Correction

Drug concentrations were determined every 3 min by measuring UV-Vis spectra in both layers for two reasons:

(a) In the aqueous phase, in order to perform scattering correction caused by precipitation or liquid–liquid phase separation (LLPS) of supersaturated solutions, formulation ingredients, or medium effects,
(b) In the organic phase, in order to correct for an overlap of the absorption with partitioned excipient spectra.

**Figure 2:**
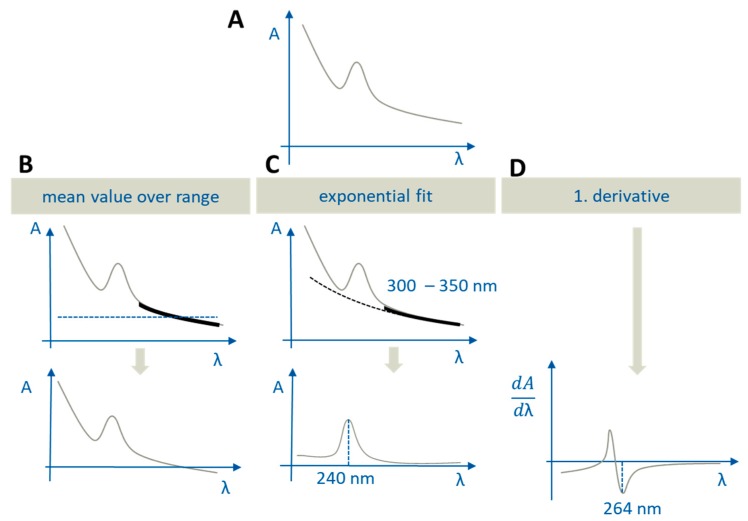
Different options to correct scattering in UV-Vis spectra: (**A**) Uncorrected spectrum; (**B**) mean value over range, black bold range: Mean value calculation (at the top) and the corrected spectrum (at the bottom); (**C**) exponential fit, black bold range 300–350 nm: Fitting function range (at the top). Ritonavir is quantified at 240 nm using the corrected spectrum (at the bottom); (**D**) first derivative and the inflection point at 264 nm, where ritonavir is quantified (at the bottom).

The implemented processing methods for correcting scattering and overlapping effects in the developed LabView^®^ application were mean values over range, mono-exponential fit, and n^th^ derivatives (Figure 2).

Mean value over range was used to correct a simple offset of the spectra. The mean absorption value over a selected wavelength range is subtracted from the origin spectra. Correcting a spectrum by a mono-exponential fit (absorption (λ) = a*e^−kλ^) describes the scattering phenomena most accurately. The processing by derivatives is very helpful in order to evaluate overlapping spectra. Additional advantages of derivatives are the loss of the intercept and a lower degree of error caused by scattering [21]. For the correction of the spectra in the aqueous phase, the mono-exponential fit was applied and in the organic phase the first derivative was formed.

The ritonavir concentration in the aqueous layer was quantified at 240 nm (pH > 5.5) and 245 nm (pH = 1.0), correcting the related spectra using the mono-exponential fit function. To determine the fitting function, the range of 300–350 nm (black bold line) was selected and the resulting function was subtracted from the original spectra. Ritonavir was determined in the 1-decanol layer based on the first derivative. The derived spectrum was subsequently evaluated at 264 nm to exclude overlapping with spectra of formulation excipients or components of the dissolution media.

#### 2.5.3. Buffer Design

For the described method, an in situ pH-shift from pH 1.0 to 5.5 and, afterwards, stepwise (increment: 0.25 pH) to 6.8 was employed mimicking the human gastrointestinal passage [22]. A suitable buffer composition or the proposed mini-scale method should have the following properties:

(a) Highly concentrated in order to minimize volume changes,
(b) Equal buffer capacities between pH = 5.5 and 6.8 [23,24],
(c) Comparable osmolarity and buffer capacity to in vivo [23].

To meet these requirements, the development of a buffer concentrate was based on McIlvaine buffer [25], as a physiological carbonate buffer was not suitable for the online dissolution test due to carbon dioxide gas development. McIlvaine buffer solutions are mixtures of a phosphate and a citrate buffer system, which facilitates comparable in vivo buffer capacities, because pK_a_-values of citrate/phosphate buffer are similar to the pK_a_-values in vivo in the entire range of physiological pH-values (Table 1). Thus, we regarded citrate and phosphate as biosimilar buffer components. Therefore, it was possible to design a dynamic pH-profile, which is described in literature [22,26].

Three steps were taken to develop a buffer composition with comparable buffer capacities at pH = 5.5 and pH = 6.8: (1) Optimization of molar ratio between citrate and phosphate to reach consistent buffer capacities between pH 5.5 and 6.8 [26], (2) maximization of buffer concentration to minimize its volume, and (3) optimization of the buffer capacity and osmolarity to human data.

#### 2.5.4. Addition of Bile Salts

To generate an in-situ biorelevant aqueous medium for the fasted state, it was necessary to add biorelevant surfactants, namely sodium-taurocholate and lecithin. In the present study, the concentrations of sodium-taurocholate and lecithin were kept at the same level as for Fasted State Simulated Intestinal Fluid (FaSSIF) or Fasted State Simulated Intestinal Fluid Version 2 (FaSSIF-V2) [26,28]. Both surfactants, sodium-taurocholate (272.3 mg) and lecithin (97.5 mg in FaSSIF or 26.0 mg in FaSSIF-V2), were solubilized in 1100 µL demineralized water. The biorelevant media formed in situ by adding 333 µL surfactant concentrate to the aqueous medium (Table 1). Three media were investigated in the present study [29]:

1. Bi-FaSSIF: pH-gradient with surfactant concentration of FaSSIF,
2. Bi-FaSSIF-V2: pH-gradient with surfactant concentration of FaSSIF-V2,
3. Buffer: pH-gradient without biorelevant surfactant.

#### 2.5.5. Selection of Organic Medium

In many studies, 1-octanol is used for generating distribution- or absorption-sink conditions [10,28,29,30,31] due to its physiochemical properties and its common use in logP determination. However, 1-octanol displays two major drawbacks for the use in our BiPHa+ model: (1) The high solubility of octanol in water (0.5 g/L), and (2) the bad odor reducing personal compliance. In contrast, 1-decanol exhibits a water solubility of 0.04 g/L [32]. Thus, decreased interaction between the organic solvent and the formulation in the aqueous medium could be expected. No evaporation is expected because of the high boiling point of 230 °C.

#### 2.5.6. Experiment Sequence

**Figure 3:**
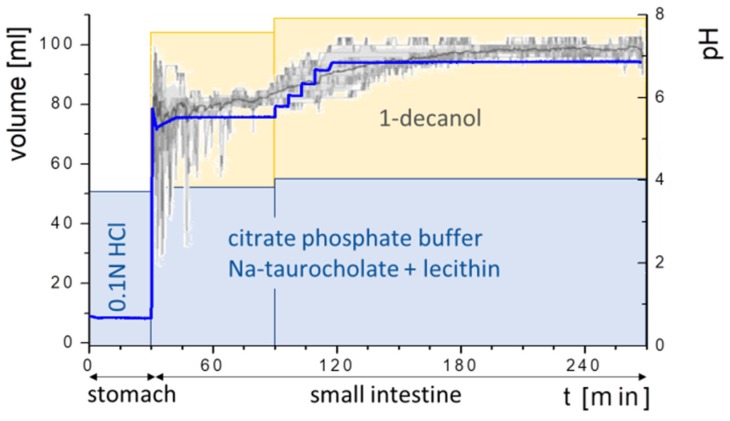
Dissolution procedure: (**stomach**) Formulation disperses for 30 min in 0.1 M hydrochloric acid; (early **small intestine**) addition of surfactant concentrate after 30 min, buffer concentrate to reach pH 5.5, and overlaying of the aqueous phase with 50 mL of 1-decanol; (late **small intestine**) stepwise pH-adjustment at 90 min to reach pH 6.8. The grey line in the background represented measured human gastrointestinal pH-profile [22].

Prior to the start of the experiments, both phases, 1-decanol and the acidic aqueous phase, were saturated with each other. The selection of transition time and pH-profile were guided by Koziolek et al. [22]. During the first 30 min, the formulation disintegrated/dispersed in 50 mL of 0.1N HCl. Subsequently, 333 µL surfactant concentrate was added to generate Bi-FaSSIF of Bi-FaSSIF-V2. In the case of the plain buffer medium, no surfactant was added. Then, pH was shifted to 5.5 by adding the buffer concentrate. The aqueous layer was covered by 1-decanol within 30 s after the pH shift. At 90 min, the pH was further increased stepwise to pH 6.8. In contrast to the reported colon arrival time of 270 min, the overall test duration was 390 min to enable a comparison to previously published data [33] (Figure 3).

### 2.6. Comparative Studies

A general purpose of the present study was to combine the advantages of a small-scale one-vessel method as a useful tool in formulation screening [13,14,34] and the proven biorelevance of the USP II and USP IV model [12,35,36]. All experiments were conducted in triplicate. Dissolution behavior was studied using three different aqueous buffer media, namely Bi-FaSSIF (M1), Bi-FaSSIF-V2 (M2), and plain buffer without surfactant (M3). The relative absorption area (A_rel_) was adjusted to a value of 0.4. The volume ratio (R_V_) of the aqueous and organic medium was kept at 1 similar to most of the published models [10]. The target concentration of the aqueous phase was calculated based on the biopharmaceutical classification system as the ratio of the highest ritonavir dose of 100 mg [37] and 250 mL, resulting in 0.4 mg/mL.

#### 2.6.1. Hydrodynamic Assessment

Hydrodynamic influences drug dissolution and precipitation as well as partitioning in the organic layer [12,17,19]. Therefore, sufficient mixing is of vital importance to avoid an unstirred water layer. Both described biphasic dissolution setups were assessed regarding their hydrodynamic [19]. The volumes of the aqueous phase and the mixing speed were set to 160 rpm for our method, while Xu et al. used 60 rpm for an USP II/USP IV combination [33]. To visualize the velocity of the water, glitter powder (density 1.33 g/cm [38] was used. Due to the high density of the glitter particles, they were assumed as worst-case precipitated particles. Photographic images were taken to illustrate the hydrodynamic in the dissolution vessel (Canon EOS 700D SLR, Tokyo, Japan at 1/320 s exposure time).

#### 2.6.2. Comparison of the BiPHa+ Test with Established USP II/IV Biphasic Methods

The reference biphasic dissolution test for the present study basically consists of a combination of an USP II and an USP IV apparatus [12,33,39]. Initially, the ritonavir drug product disintegrates/dissolves in 41 mL SGF (pH = 1.6) in the flow-through cell (USP IV) at 5 mL/min for 30 min in a closed loop [33]). Later the closed loop is coupled with the USP II including 200 mL of FaSSIF-V2 and 200 mL of octanol. Both phases were saturated with each other before the experiment starts. The biphasic dissolution test runs for 6 h. The quantification in the organic layer was conducted by UV-Vis measurement using the second derivative, excluding interferences from formulation excipients or dissolution medium. Aqueous samples were quantified by HPLC after centrifugation [33]. The published data were extracted using Engauge Digitizer 10.9 [40].

A comparison of the dissolution test setup for the BiPHa+ and the USP II/IV biphasic setup is given in Table 2. The BiPHa+ setup was parameterized to keep the surface-volume ratio (A/V), the volume-volume ratio (V/V), and the ritonavir concentration in the aqueous phase (C_BCS_) similar.

### 2.7. Development of an Advanced Kinetic Model

A kinetic model was established guided by the work of Locher et al. [13]. It is based on ordinary differential equations (ODE), which describe the fraction of dissolved drug in both phases resulting from the disintegration of the ritonavir-containing ASD, API dissolution, and partitioning into the organic phase. These ODEs were numerically solved by the Runge–Kutta approximate method. The ODEs were iteratively calculated by a self-programmed LabView^®^ application. To describe and explain the concentration-time profiles resulting from BiPHa+ experiments appropriately, the kinetic model was advanced by introducing additional phase separation processes in the aqueous phase: (1) The formation of API-rich nano-droplets via liquid–liquid phase separation (LLPS) as described by Xu et al. [33] for the same ritonavir-ASD, and (2) the process of transformation of the nanosized droplets to smaller particles, whereas the API remained amorphous [41]. Further details are given in Section 3.5.

## 3. Results and Discussion

### 3.1. Physiochemical Characterization

The physicochemical characterization of ritonavir is given in Table 3:

### 3.2. Buffer Development

**Figure 4:**
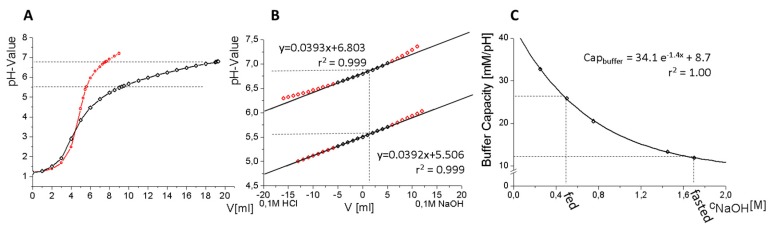
(**A**) pH Profile of 50 mL 0.1 N HCl by adding potassium-citrate and tri-potassium-phosphate; red line represents different buffer capacities, and black line represents similar buffer capacities between pH 5.5–6.8. Buffer capacities are similar if the slope of the titration curves at pH = 5.5 and 6.8 are similar, which is the case for the black-colored curve; (**B**) titration profile of adjusted buffer at 5.5 and 6.8 to determine the buffer capacity at these levels. The adjusted buffers were titrated by using either 0.1 M NaOH or HCl. The slopes represent the buffer capacity at pH = 5.5 and pH = 6.8 (black line); (**C**) optimization of buffer capacity by adding sodium hydroxide to the concentrate to reach fasted and fed conditions at pH = 6.8.

In the first step, a pH-profile was generated by adding increments of 1 mL of different ratios of potassium phosphate and potassium citrate concentrate (molar K_3_PO_4_/K_3_C_6_H_5_O_7_ ratios are between 0.2/0.3 and 0.05/0.4) to 50 mL 0.1 N HCl (Figure 4A). The generated pH-profile provides information about the buffer capacity. The slopes of titration profiles at pH 5.5 and 6.8 were assumed as surrogate parameters for the buffer capacity. If the slopes at pH 5.5 and 6.8 are comparable with each other, the buffer capacity will also remain equal (Figure 4A,B).

In the second step, the adjusted buffers were titrated by 0.1 M NaOH and 0.1 M HCl to verify the buffer capacity at pH = 5.5 and 6.8. The slopes of the received titration profile represented the buffer capacity. A molar ratio between phosphate (0.15M K_3_PO_4_) and citrate (0.35M K_3_C_6_H_5_O_7_) of 0.429 was found to be the optimum resulting in equal buffer capacities at pH = 5.5 and 6.8 during the intestinal phase of the dissolution experiment. Higher concentrated citrate and phosphate with the same molar ratio resulted in the same buffer capacity by adding less volume. The final concentrations of both buffer salts were 0.225 M for potassium citrate and 0.525 M for tri-potassium phosphate. Higher concentrations were not possible because of solubility limitations.

In the last step, buffer capacity and added volume were optimized. Different amounts of sodium hydroxide were added to the concentrate to decrease both buffer capacities and the required addition volume to achieve pH 5.5 or 6.8 (Figure 4C). The buffer capacities can be described by an exponential fit (Figure 4C), so that the desired composition can be calculated. With the optimized buffer concentrate, it is feasible to generate aqueous media of various pH with biorelevant buffer capacity and osmolarity [28], and can further be used to simulate an altered gastric stomach by adjusting the pH to 4.5. The proposed buffer compositions are shown in Table 4.

### 3.3. Hydrodynamic Assessment

**Figure 5:**
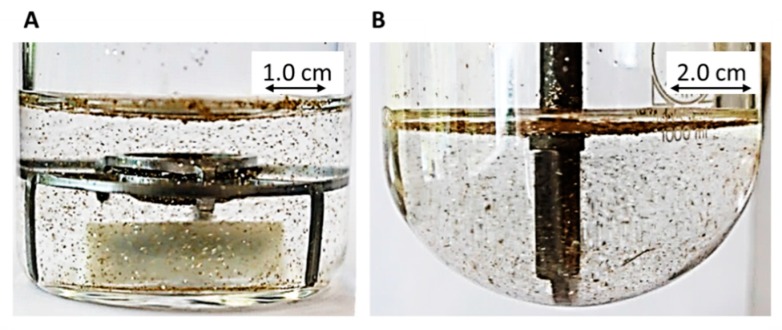
Hydrodynamics in (**A**) BiPHa+ apparatus (50 mL at 160 rpm), and (**B**) USP II apparatus (200 mL at 60 rpm).

In both cases, the dissolution vessel showed optimal hydrodynamic under the assessed mixing conditions, which is visualized by the distribution of glitter particles (Figure 5). Neither an unstirred water layer nor a funnel was formed. This can be explained by the turbulent movement of the water in both vessels caused by the triangular stir bar or the paddle. Due to the high mixing performance of glitter, no unstirred water layer was expected for the ritonavir-containing droplets (precipitates) and dissolved ritonavir, which is crucial to achieve homogeneous and comparable partitioning processes.

### 3.4. Comparison of All Dissolution Profiles

**Figure 6:**
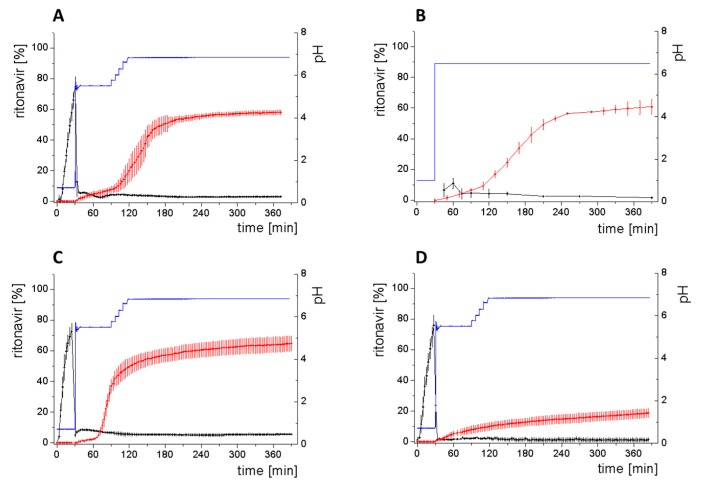
Biphasic dissolution experiments in (**A**) Bi-FaSSIF-V2 medium (BiPHa+), (**B**) Model of Xu et al. [33], (**C**) Bi-FaSSIF-V1 medium (BiPHa+), (**D**) Buffer without surfactant (BiPHa+); API concentration in the 1-decanol layer (red line, mean value and standard deviation); dissolved ritonavir in the aqueous medium (black line, mean value and standard deviation); pH values (blue line). All experiments were done in triplicate (*n* = 3).

The different ritonavir concentration–time profiles using Bi-FaSSIF, Bi-FaSSIF-V2, and plain buffer are shown in Figure 6. These three fasted state media including pH-change were compared to the results of Xu et al. [33], where ritonavir drug products were tested in 41 mL gastric fluid and subsequently diluted with FaSSIF-V2. No quantification of the drug during the first 30 min was performed. In the present study, the amount of ritonavir was quantified during the entire experiment in both layers. In the gastric stage, approximately 75% of the drug was released into the aqueous phase after 30 min for all three investigated media (Figure 6) demonstrating reliable hydrodynamic conditions. Subsequent to the gastric period, buffer and surfactant were added. At intestinal dissolution stage, the dissolution rapidly decreased due to the lower solubility of ritonavir at pH 5.5. Results from the BiPHa+ test containing biorelevant surfactant and the method of Xu et al. [33] showed a comparable concentration range in the aqueous phase. At 50 min, a supersaturation peak of 10% (50 µg/mL) occurred in all setups containing biorelevant surfactant, which is probably the result of the ongoing dissolution process of the remaining ritonavir-containing ASD. Subsequently to this peak value, the concentration slowly decreased to 3% in Bi-FaSSIF-V2, 5% in Bi-FaSSIF-V1, 2% in plain buffer medium, and 2% in the method of Xu et al. [33] (HPLC determination) at the end of the experiment. Very slight supersaturation was observed in the plain buffer setup and the concentration remained at the same level of 2% (Figure 6D). The Bi-FaSSIF-V2 experiment was additionally evaluated by HPLC. Ritonavir concentrations are comparable with results from online UV measurement, and in agreement with values reported by Xu et al. [33] (Table 5). A one-way analysis of variance (ANOVA) was preformed to evaluate the statistical significance on a difference between the two analyzing methods. A significance level of α = 0.05 was assumed. A p-value of 0.87 was the result, which indicates that the quantification method did not influence the resulting concentration. Consequently, the implemented online UV quantification with scattering-correction for the BiPHa+ method provides reliable results.

The dissolution experiment in Bi-FaSSIF-V2 (Figure 6A) and Bi-FaSSIF-V1 (Figure 6C) resulted in similar sigmoidal ritonavir concentration profiles for the organic phase as the one reported by Xu et al. [33] (Figure 6B). The partitioning of ritonavir was at a lower level in the early intestinal stage. During the course of dissolution, the partition rates into the organic phase increased. The inflection points occurred at earlier time points with increasing surfactant concentration in the dissolution medium. Furthermore, the slopes at these points increased with higher surfactant concentration. Hence, the partitioning rate of ritonavir into the organic phase increased. A plateau at 60% drug concentration was reached in all three dissolution media. In contrast, the ritonavir concentration in the organic phase by using plain buffer without biorelevant surfactants steadily increased without any change of the overall low partitioning rate and reached a maximum ritonavir concentration of only 19% at the endpoint of the dissolution experiment.

The drug concentration profile of the 1-decanol phase demonstrated its function as absorption compartment. The partitioning of ritonavir into the organic layer was much more pronounced in biorelevant medium, whereas without bile salts and lecithin, the redissolution and partitioning rate into the organic layer were dramatically reduced. Interestingly, the amount of dissolved ritonavir in the aqueous phase was nearly the same in all cases. This observation supported the hypothesis from Taylor et al. [43], that API-rich nano droplets from liquid–liquid phase separation rapidly equilibrate with the dissolved drug in the aqueous medium. The nano-roplets serve as a reservoir, and enable the replenishment of dissolved API, that partitioned into the organic phase [43]. The formation of ritonavir-nano-droplets in biorelevant media from a ritonavir-ASD is also reported by Xu et al. [33]. Therefore, the process of redissolution of the API from the nano-droplets is of great importance for the performance of amorphous solid dispersions and is further described in Section 3.5.

### 3.5. Kinetic Model

As reported in Section 3.4, the dissolution in biorelevant media (Bi-FaSSIF-V1/V2) resulted in a sigmoidal partitioning profile into the organic phase, whereas the ritonavir concentration by using plain buffer without surfactants steadily increased without any change of the slow partitioning rate. For a better kinetic description and explanation of resulting organic concentration–time profiles, the kinetic model of Locher et al. [13] for crystalline BCS II APIs was advanced towards an ASD by introducing additional phase separation processes in the aqueous phase as described in the following section.

#### 3.5.1. Physical Explanations of the Complex In Vitro Dissolution Kinetics for Ritonavir

**Figure 7:**
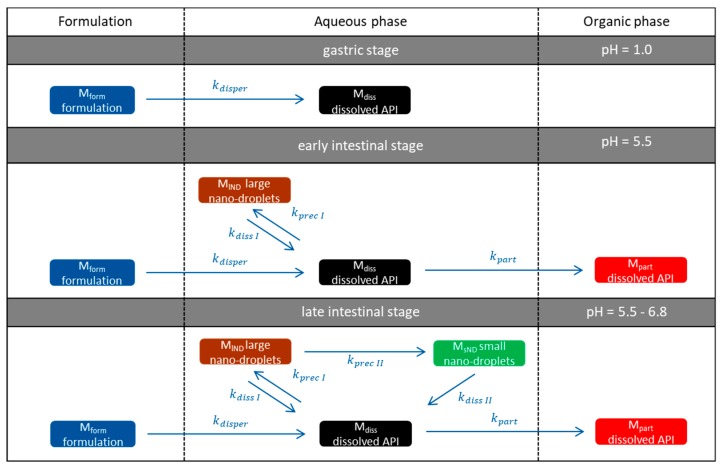
Schematic illustration of the five-compartment model to describe the proposed kinetic processes occurring in the BiPHa+ dissolution from dispersing of the ritonavir-amorphous solid dispersion (ASD) formulation to ritonavir partitioning into the organic (1-decanol) layer.

**Figure 8:**
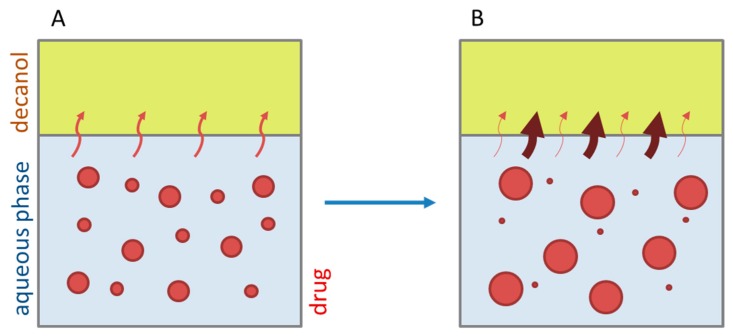
Schematic biphasic dissolution behavior of a ritonavir ASD: (**A**) In the course of the dissolution process, different sizes of nano-droplets (red dots) are formed via liquid–liquid phase separation (LLPS). Dissolved ritonavir partitions into the organic layer (red arrows). (**B**) After a while, larger and smaller ritonavir nano-droplets are generated because of Ostwald ripening, re-dissolution, and partitioning. A higher re-dissolution rate of ritonavir from the small nano-droplets results in an increased partitioning rate (red bold arrows). In contrast, a lower re-dissolution rate of ritonavir from large nano-droplets ends up in a decreased partitioning rate (narrow red arrows).

The kinetic profile obtained from of the BiPHa+ dissolution experiment can be divided into three different stages as illustrated in Figure 7:

1. Gastric stage—The ritonavir-containing ASD was tested for 30 min at pH 1.0 without the 1-decanol absorption layer. Upon contact with the dissolution medium the ritonavir-ASD disperses, API and excipients dissolve simultaneously [44], and a solution is generated. Dispersing of the ASD and dissolution of ritonavir are assumed as one irreversible step. Furthermore, it can be assumed that drug and polymer dissolve simultaneously for the given ritonavir-ASD at a drug load of 15% as demonstrated by Indulkar et al. [44].
2. Early intestinal stage—After covering the aqueous layer with 1-decanol and changing the pH from 1.0 to 5.5, a 2-compartment model for the simulated early intestine was applied (Figure 7). Liquid–liquid phase separation (LLPS) occurs from the solution, when crystallization is slow [41] and the amorphous solubility of the API is exceeded leading to the formation of nanosized API-rich nano-droplets [33,45].
  In the present kinetic model, the formation of the nano-droplets refers to the generation of the large nano-droplets as shown in Figure 8 and Figure 7. The ritonavir-rich nano-droplets provide a reservoir replenishing dissolved ritonavir that irreversibly partitioned into the organic layer [33,43,46,47].
3. Late intestinal stage—At the timepoint, where the partitioning of ritonavir into the organic phase substantially increases, the re-dissolution rate of ritonavir from the nano-droplets into the aqueous phase increases, while the particle size of the small nanoparticles decreases [48,49,50]. Furthermore, the particle size reduction can be further driven by Ostwald ripening of the nano-droplets [48] as also reported for nano-emulsions, which exhibit a typical size of 20 to 200 nm [51]. This is in the same size range as reported for the same ritonavir-ASD in a previous study [41,48]. The resulting dispersion formed in buffered aqueous medium contained particles at a mean diameter of approximately 60 nm in a monomodal particle size distribution [48]. Briefly, during the thermodynamically driven process of Ostwald ripening molecules on the surface of energetically unfavorable small particles (e.g., the API molecules from the nano-droplets) tend to detach from the particles and diffuse into the solution as free molecules generating a supersaturated solution. At the stage, where supersaturation is achieved, the free molecules have the tendency to condense on the surface of larger particles, or in our case, larger nano-droplets. Therefore, the smaller nano-droplets continue to shrink, while larger nano-droplets continue to grow as illustrated in Figure 8. While ritonavir is continuously removed by partitioning into the organic phase, the size of small nano-droplet decreases until they disappear. The continuously shrinking nano-droplets are described by forming small nano-droplets in the kinetic model (Figure 7). It is assumed that large nano-droplets and small nano-droplets lead to different kinetic properties in terms of the ritonavir dissolution from the nano-droplets due to their difference in particle size (Supplementary Materials).

Because the surface energy has a great impact on the re-dissolution rate, the re-dissolution rate and change in particle size distribution is further increased in the biorelevant media [48,49]. For this reason, the partitioning of ritonavir into the organic layer was much more pronounced in biorelevant medium leading to a sigmoidal concentration time curve (Figure 6A–C), whereas re-dissolution and partitioning into the organic layer are dramatically reduced without bile salts and lecithin (Figure 6D). Remaining larger nano-droplets can merge by coalescence, as most likely they are not stable in the surfactant-free buffer medium. Consequently, the particle size distribution of the droplets can move further towards particle sizes in the lower micrometer scale during the course of the dissolution experiment [41,48].

The concentration of ritonavir in 1-decanol reaches a plateau at about 120 min in Bi-FaSSIF-V2 (Figure 6A), 270 min in FaSSIF-V2 (Figure 6B), and 180 min in Bi-FaSSIF (Figure 6C) indicating a reduced concentration of free ritonavir in the aqueous medium. The presence of mainly very large nano-droplets or even coalesced droplets led to only very limited ritonavir dissolution into the aqueous medium. In the surfactant-free buffer medium, these very large particles permitted only very slow ritonavir dissolution resulting in turn in an overall very low partition rate into the organic layer (Figure 6D).

Based on the assumptions provided above, five compartments (M) and six related rate constants (*k*) were introduced in the model to describe the concentration–time profiles kinetically. Each compartment was assigned to certain rate constants, which were negative, when the described process decreased the amount of ritonavir in the corresponding compartment:

1. M_form_: The undissolved ritonavir-ASD formulation,
2. M_diss_: Dissolved ritonavir in the aqueous phase,
3. M_lND_: Ritonavir-rich nano-droplets,
4. M_sND_: Small, shrunken ritonavir-rich nano-droplets,
5. M_part_: Dissolved ritonavir in the organic phase.

Further details are given in Figure 7 and Table 6.

#### 3.5.2. In-Silico Model Fitting of Ritonavir In Vitro Dissolution and Partitioning Kinetics

**Figure 9:**
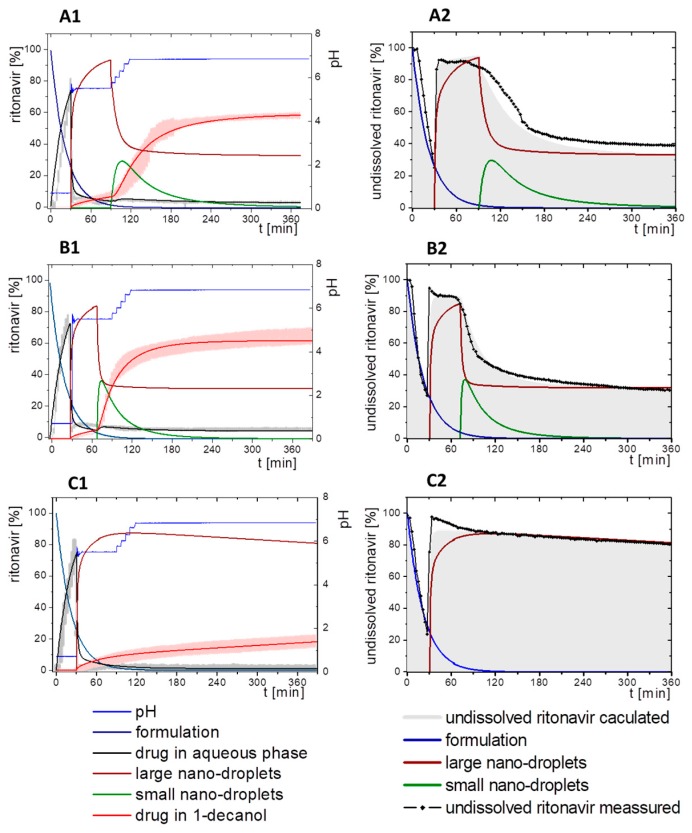
Model fit of four or five compartments compared to the experimental data (**A1**–**C1**) and mass balance of undissolved ritonavir portions (**A2**–**C2**). (**A1**) in Bi-FaSSIF-V2 including small nano-droplets (green line), (**B1**) in Bi-FaSSIF-V1 including small nano-droplets (green line), (**C1**) in surfactant-free buffer medium without a nano-droplet transformation. (**A2**) in Bi-FaSSIF-V2 including small nano-droplets (green line), (**B2**) Bi-FaSSIF-V1 including small nano-droplets (green line), (**C2**) in surfactant-free buffer medium without a nano-droplet transformation.

The differential equations as well as the corresponding graphs describing the vitro dissolution and partitioning kinetics are represented in Table 6. The iterative fittings of M_diss_ (Equations (5), (6), and (9)) and M_part_ (Equations (8) and (12)) were compared with the amount of ritonavir in the aqueous and the organic medium. The amount of M_form_ (Equation (4)), M_lND_ (Equations (7) and (10)), and M_sND_ (Equation (11)) directly resulted from the calculation. The rate constants (Table 7) and kinetic profile (Figure 9) from the experiments in three different dissolution media were calculated accordingly. Figure 9 illustrates the kinetic profiles of the five compartments fitted with regard to the dissolved ritonavir (A1–C1). Figure 9(A2–C2) represents the mass balance of the unresolved ritonavir proportions. For this purpose, the measured undissolved ritonavir proportions (black line) were compared with the calculated undissolved proportions (grey area). The calculated undissolved ritonavir includes ritonavir in the formulation (M_form_), small and large nano-droplets (M_lNM_, M_sND_).

The process of dissolution started in the gastric medium within the first 30 min with dispersing of the ritonavir-ASD formulation (Equation (4)) immediately followed by the dissolution of the ritonavir. The dispersing of the ritonavir-ASD formulation and the ritonavir dissolution from the compartment M_form_ was described by a first-order kinetic with *k_diss_* as rate constant (Equations (4) and (5)). Dispersion and dissolution from the ritonavir-ASD were assumed to be identical [44], because the API was molecularly dissolved in the polymer in contrast to the case of crystalline API described by Locher et al. [13]. If the polymer dissolves, the drug will also dissolve immediately. Approximately 75% of the ritonavir was dissolved at the end of the gastric stage (Figure 9).

During the early intestinal stage, the ritonavir-ASD was further dispersed. Because of the strongly pH-dependent solubility of ritonavir, the formation of the ritonavir-rich nano-droplets (M_lND_) occurred directly after pH-adjustment (Table 6). The organic absorption layer was added 30 s after pH-change to 5.5. The compartment of dissolved ritonavir (M_diss_) was complemented with that of the ritonavir-rich nano-droplets (M_lND_) and the ritonavir absorption compartment (M_part_) of 1-decanol (Equations (6)–(8)). The partitioning of dissolved ritonavir into the organic phase, (M_part_) was followed by an irreversible first-order kinetic. In the case of Bi-FaSSIF-V1 and Bi-FaSSIF-V2, the kinetic model in the early intestinal stage comprises a four-compartment model that accurately describes the experimental data between 30 and 80 min as well as between 30 and 65 min, respectively. After these periods, the partitioning rate strongly increased, leading to a sigmoidal distribution profile (Figure 6 and Figure 8). On the other hand, the absence of a sigmoidal partitioning profile in surfactant-free buffer medium confirmed the early intestinal model for the whole experimental period (Figure 9C).

Ritonavir-ASD dispersion and ritonavir dissolution into the compartment M_diss_ as well as the partitioning process into the organic phase (M_part_) were assumed to be irreversible. In contrast, re-dissolution of ritonavir from large nano-droplets (M_lND_) into the compartment M_diss_ was considered as reversible. Furthermore, the model was able to describe the remaining ritonavir in the formulation compartment (M_form_) after changing the pH from 1.0 to 5.5, and further to 6.8, which is described by a first order dissolution model, because the polymer dissolved pH-independently and the polymer matrix properties controlled the dissolution. This means that the active ingredient dissolves independently of the outer environment but depends on the polymer [44].

The generation of the ritonavir-rich nano-droplets (M_lND_) and the dissolution of the ritonavir into M_diss_ are limited by the residual dissolved ritonavir at the end of the experiment, which is expressed by the term (*sat −* M_diss_) and (M_diss_ *− sat*) (Equations (6) and (7)). *Sat* values represent the measured ritonavir concentration in the aqueous phase at the end of the test [13]. Supersaturation followed by the formation of ritonavir-rich nano-droplets (M_lND_) occurred at concentrations of freely dissolved ritonavir of higher than *sat*. The scaling factor was negative for the dissolution equation and positive for the precipitation equation (Equation (7)).

In the late intestinal stage, all rate constants and model descriptions from early intestinal stage remained unchanged in further calculations (Table 7). The model was extended by a conversion process of ritonavir-rich nano-droplets generated from liquid–liquid phase separation (LLPS) during precipitation of dissolved ritonavir (M_lND_) into smaller ritonavir-rich nano-droplets (M_sND_) in Bi-FaSSIF and Bi-FaSSIF-V2, and described the changing re-dissolution and subsequently partitioning rate (Equations (10) and (11)). The presence of smaller nano-droplets (M_sND_) can physically explain the enhanced dissolution rate, which became the new rate limiting step (Table 7). Neither a direct formation of large nano-droplets in the M_lND_ compartment from small nano-droplets (M_sND_) nor precipitation from dissolved ritonavir into small nano-droplets (M_sND_) occurred (Equations (10) and (11)). The expressions (*max* − M_lND_) and (M_lND_ *− max*) (Equations (10) and (11) were introduced to scale the extent of the larger nano-droplets (M_lND_), which underwent particle size reduction and contributed to partitioned ritonavir (see Section 3.5.1). *Max* values represented the number of large particles, which were not able to transform in smaller particles. The scaling factor is negative for the M_lND_ equation and positive for the M_sND_ equation. Thus, the amount of M_lND_ decreased and the amount of M_sND_ increased. *Max* values were result of the model fitting: *max* = 32% in Bi-FaSSIF-V1 and *max* = 35% in Bi-FaSSIF-V2.

The partitioning of ritonavir into the organic phase (M_part_) was described by a second-order kinetic model (Equation (12)). A second-order model described the partitioning into the organic phase, which depended on the amount of dissolved ritonavir in the aqueous phase (M_diss_) as well as the amount of ritonavir-rich nano-droplets (M_sND_). This assumption took two considerations into account: (1) The fast re-dissolution rate of small nano-droplets (M_sND_), and (2) the plateau in the organic phase at the end of the experiment (Figure 9). A plateau was reached, when the number of small nano-droplets (M_sND_) decreased. The larger nano-droplets in the M_lND_ compartment dissolved very slowly and did not substantially contribute to the partitioning of ritonavir in the organic phase (M_part_). The extent and rate of plateau formation depends on the lecithin/taurocholate ratio [52].

The obtained rate-constants from the simulation results are listed in Table 7. The models matched very well with the experimental data (Figure 9). In all simulations, the same dispersion/dissolution rate (k_disper_) constant was determined during the first 30 min in the gastric part.

Immediately after shifting the pH to 5.5 and covering the aqueous layer with 1-decanol, LLPS occurred from the solution leading to the formation of nanosized API-rich nano-droplets (M_lND_). The rate of redissolution of ritonavir from these nano-droplets (M_lND_) was very slow (k_prec1_ = 0.009 min^−1^) and represented the rate limiting step in all three tested dissolution media. Dispersing of the formulation, formation of API-rich nano-droplets, and re-dissolution had the same rate constants in all experiments. Consequently, they were hardly influenced by the dissolution medium (Table 7).

The Bi-FaSSIF and the Bi-FaSSIF-V2 dissolution medium led to the same partitioning rate (k_part_ = 0.015 min^−1^) during the period of 30 to 80 min and 30 to 65 min, respectively. In this period, no transformation into small nano-droplets (M_sND_) occurred.

In contrast, the calculated partitioning rate in the plain buffer (k_part_ = 0.023 min^−1^) during the early intestinal time period starting at 30 min was little faster (Table 7) resulting in an increased onset of dissolved ritonavir in the organic phase (Figure 9). The difference in the kinetics can be explained by the presence of surfactants in the two biorelevant media, which could lead to a higher affinity of the API in the aqueous phase, and potentially influence the 1-decanol/buffer interface [53].

The nano-droplet properties in the plain buffer medium did not change until the end of the experiment.

Ritonavir-rich nano-droplets (M_lND_) started to convert into small nano-droplets (M_sND_) at 65 min (Bi-FaSSIF) and 80 min (Bi-FaSSIF-V2), which demonstrated faster dissolution rates. During dissolution, decreased nano-droplet size led to an increased solubility with time and a shorter total dissolution time. Consequently, the generated small nano-droplets (M_sND_) displayed different kinetics for the ritonavir dissolution compared to larger nano-droplets (M_lND_), especially in the presence of surfactants (Figure 9A,B). Higher surfactant concentrations potentially resulted in a higher wettability of the nano-droplets, resulting in a faster shrinking of small nano-droplets, and a faster re-dissolution rate of the ritonavir from the small nano-droplets (M_sND_) [49,51]. The re-dissolution rate (k_dissII_) constant increased to 0.04 min^−1^ and 0.05 min^−1^, respectively, and became the new rate limiting step (Table 7). It is important to emphasize that the partitioning (k_part_) rate also increased to 0.45 min^−1^ and 0.55 min^−1^. This led to the conclusion that the kinetic stability of small nano-droplets (M_sND_) strongly decreased caused by an increased surface energy [48,49], which led to a faster re-dissolution and partitioning process. The decreased kinetic stability was expressed in the kinetic model by a second-order partitioning model dependent on the number of small nano-droplets (M_sND_) and the dissolved ritonavir concentration (M_diss_).

In sum, the present kinetic modeling was able to describe the dissolution and partitioning of ritonavir in the BiPHa+ dissolution test on the basis of the dispersion and dissolution behavior of a ritonavir-ASD via API-rich nano-droplets generated by LLPS. The model considered the impact of nano-droplet size on ritonavir dissolution and partitioning as well. Furthermore, the model was able to quantify the amount of growing (M_lND_) and shrinking (M_sND_) particles by the Ostwald ripening. The described mechanism provides an additional aspect of the reservoir effect of API-rich nano-droplets by LLPS, which in turn, can explain the potential of ASDs to increase the dissolution rate of the incorporated API and to enhance its bioavailability.

## 4. Conclusions

A fully automated, small-scale, one-vessel biphasic dissolution setup (BiPHa+) was developed, which was characterized by the volume ratio of aqueous and organic phase, the ratio of absorption area to aqueous volume, as well as the concentration of ritonavir in the aqueous phase. UV- quantification in both layers was suitable to determine the drug concentration despite the appearance of scattering or overlapping spectra. One major advantage of BiPHa+ apparatus is the automation of critical analytic parameters such as dispensing of liquids and pH adjustment. The control of hydrodynamics is realized by triangle magnetic stirrers creating a turbulent movement of the aqueous phase. The one-vessel dissolution method is able to generate comparable data to the biphasic method of Xu et al. [33] consisting of a combination of USP apparatus II and IV, which has already demonstrated in vivo relevance [36,39].

Both media Bi-FaSSIF and Bi-FaSSIF-V2 led to complex partitioning profiles, while the partitioning rate of the experiment in surfactant-free medium was more homogeneous and reached a much lower level of distributed drug in the organic phase. Thus, constitution and redissolution were significantly influenced by biorelevant surfactants.

In order to get a deeper insight into these complex behaviors, a kinetic model was implemented including the transformation of the nano-droplets, which was necessary to explain the sigmoidal distribution kinetics profile. The kinetic model described the setup mathematically, and parametrized the processes of disintegration, dissolution, precipitation as nano-droplets, re-dissolution, and absorption. As a result, the redissolution step was the time limiting step in all cases. Ostwald ripening of nano-droplets is proposed as a new effect, which can possibly influence the drug dissolution from an ASD. Beside supersaturation, an improved redissolution of precipitated API also provides an essential contribution to enhanced drug dissolution from an ASDs.

## Figures and Tables

**Table 1:** Overview of pKa values comparing McIlvaine-buffer and physiological conditions [24,26,27].

Component	McIlvaine		Human		
	Citrate	Phosphate	Carbonate	Phosphate	Organic Acid
		2.2		2.2	
	3.1		3.5		
pKa	4.8				≈5
	6.4		6.4		
		7.2		7.2	
		12.2	10.3	12.2	

**Table 2:** Dissolution setup parameters and comparison.

	BiPHa+	USP II/IV Biphasic Method [33]
Radius	2.5 cm	5.0 cm
$\frac{A_{interface}}{V_{aqueous}}$	$\frac{20{\mathrm{cm}}^{2}}{50{\mathrm{cm}}^{3}}=0.4{\;\mathrm{cm}}^{-1}$	$\frac{80{\mathrm{cm}}^{2}}{240{\mathrm{cm}}^{3}}=0.33{\;\mathrm{cm}}^{-1}$
$\frac{V_{organic}}{V_{aqueous}}$	$\frac{50{\mathrm{cm}}^{3}}{50{\mathrm{cm}}^{3}}=1$	$\frac{200{\mathrm{cm}}^{3}}{241{\mathrm{cm}}^{3}}=0.8$
$C_{\mathrm{BCS}}$	$1.0\times {\;C}_{\mathrm{BCS}}=0.4\;\frac{\mathrm{mg}}{\mathrm{mL}}$	$1.0\times {\;C}_{\mathrm{BCS}}=0.4\;\frac{\mathrm{mg}}{\mathrm{mL}}$
Mixing	Magnetic stirrer	Paddle
(Biphasic) Vessel	Cylindric vessel	USP II vessel

**Table 3:** Physicochemical characterization of ritonavir pKa values, solubility (s), concentration where liquid-liquid phase separation occurs (LLPS) and LogP.

Parameter	Value
pKa 1	1.9
pKa 2	2.5
s (0.1N HCl) [µg/mL]	382.8
s (6.8N Buffer) [µg/mL]	0.96
s (FaSSIF-V2) [µg/mL]	4.3
LLPS [µg/mL]	40 [33]
s (1-decanol) [mg/mL]	23.9
Log P	4.3 [42]

**Table 4:** Proposed buffer concentrate composition in the fasted for a dynamic pH-profile comparing to biorelevant media and necessary volumes of buffer concentrate to a 0.1 M HCl.

Composition		dV_pH5.5_ [mL]	dV_pH6.8_ [mL]	Capacity [mM/pH]	Osmolarity [mosmol/L]
K-Citrate	0.525 M	2.12	2.54	11.9	245–257
K-Phosphate	0.225 M				
NaOH	1.7 M				
FaSSIF-V1				12	270
FaSSIF-V2				10	180

**Table 5:** Comparing mean dissolution endpoint concentrations and standard deviation (SD, *n* = 3) of ritonavir concentration after 6 h under Bi-FaSSIF-V2 conditions.

	UV-Vis	HPLC	Xu et al. [33]
Organic layer	58.1% ± 1.5%	57.6% ± 5.1%	60% ± 9.1%
Aqueous Layer	3.0% ± 0.3%	2.1% ± 0.4%	~2.0% (SD not available)

**Table 6:** Schematic representation of the differential equations to describe the to describe the proposed kinetic processes in the five-compartment model occurring in the BiPHa+ dissolution of a ritonavir-containing ASD (ritonavir-ASD) from initial formulation dispersion to final ritonavir partitioning into the organic layer (1-decanol).

**Gastric Stage—Model for dispersing of the formulation and dissolution of ritonavir at pH = 1.0**			
M_form_: undissolved ritonavir in the ritonavir-ASD formulation			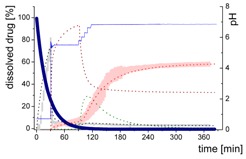
$\frac{dM_{form}}{dt}=$ $-k_{disper}\cdot M_{form}$	(4)	- Simultaneous dispersing of the formulation and dissolution of ritonavir	
**M_diss_:** dissolved ritonavir in the aqueous phase			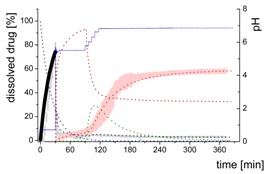
$\frac{dM_{diss}}{dt}=+k_{disper}\cdot M_{form}$	(5)	+ Simultaneous formulation dispersion and dissolution of ritonavir	
**Early intestinal stage—Simple precipitation-absorption model after initial pH-shift to 5.5**			
**M_diss_:** dissolved ritonavir in the aqueous phase			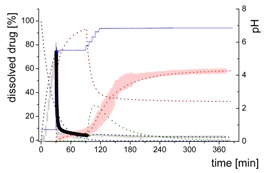
$\frac{dM_{diss}}{dt}=+k_{disper}\cdot M_{form}-k_{prec\;I}\cdot M_{diss}\cdot \left(M_{diss}-sat\right)+k_{diss}\cdot M_{lND}\cdot \left(sat-M_{diss}\right)-k_{a}\cdot M_{diss}$	(6)	+ Simultaneous dispersion and dissolution - Precipitation of ritonavir as ritonavir-rich nano-droplets (normalized by saturation solubility) + Re-dissolution of ritonavir-rich nano-droplets (normalized by saturation solubility) - Absorption of ritonavir by organic phase	
M_lND_: Large nano-droplets in the aqueous phase			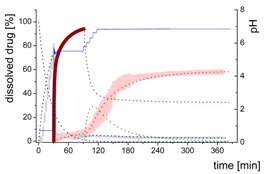
$\frac{dM_{prec\;I}}{dt}=+k_{prec\;I}\cdot M_{diss}\cdot \left(M_{diss}-sat\right)-k_{diss\;I}\cdot M_{lND}\cdot \left(sat-M_{diss}\right)$	(7)	+ Precipitation of dissolved ritonavir as ritonavir-rich nano-droplets - Re-dissolution of ritonavir-rich nano-droplets	
M_part_: Compartment: dissolved ritonavir in the 1-decanol phase			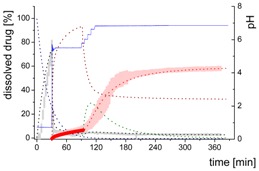
$\frac{dM_{part}}{dt}=$ $+k_{part}\cdot M_{diss}$	(8)	+ Absorption of dissolved ritonavir by the organic phase	
**Late intestinal stage—Precipitation-absorption model including a nano-droplet transformation after a stepwise pH-shift to 6.8**			
**M_diss_:** dissolved ritonavir in the aqueous phase			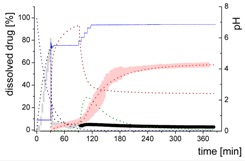
$\frac{dM_{diss}}{dt}=+k_{disper}\cdot M_{form}-k_{prec\;I}\cdot M_{diss}\cdot \left(M_{diss}-sat\right)+k_{dissI}\cdot M_{lND}\cdot \left(sat-M_{diss}\right)+k_{diss\;II}\cdot M_{sND\;-k_{part}\cdot M_{diss}\cdot \left(M_{sND}\right)}$	(9)	+ Simultaneous formulation dispersion and dissolution of ritonavir - Precipitation of dissolved ritonavir as ritonavir-rich nano-droplets + Re-dissolution of ritonavir-rich nano-droplets + Re-dissolution of small ritonavir-rich nano-droplets - Absorption of dissolved ritonavir by the organic phase	
M_lND_: Large nano-droplets in the aqueous phase			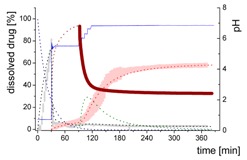
$\frac{dM_{lND}}{dt}=+k_{prec\;I}\cdot M_{piss}\cdot \left(M_{diss}-sat\right)-k_{diss\;I}\cdot M_{lND}\cdot \left(sat-M_{diss}\right)-k_{prec\;II}\cdot M_{lNM}\left(M_{lND}-max\right)$	(10)	+ Precipitation of dissolved ritonavir as ritonavir-rich nano-droplets - Re-dissolution of ritonavir-rich nano-droplets - Transformation of large nano-droplets into small ritonavir-rich nano-droplets (normalization to the amount of large nano-droplets)	
M_sND_: Small nano-droplets in the aqueous phase			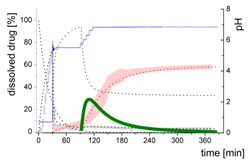
$\frac{dM_{prec\;II}}{dt}=+k_{prec\;II}\cdot M_{lND}\cdot \left(M_{lND}-max\right)-k_{diss\;II}\cdot M_{sND}$	(11)	+ Transformation of ritonavir-rich nano-droplets into small nano-droplets (normalization to the amount of large nano-droplets) - Faster re-dissolution of small ritonavir-rich nano-droplets	
M_part_: dissolved ritonavir in the 1-decanol phase			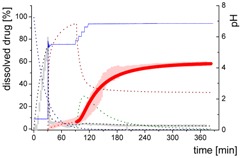
$\frac{dM_{part}}{dt}=+k_{part}\cdot M_{diss}\cdot \left(M_{sND}\right)$	(12)	+ ritonavir partitioning into the organic layer during transformation process	

**Table 7:** Estimated rate constants of three BiPHa+ settings. Bold values characterize the rate limiting steps.

Typ	Bi-FaSSIF-V2			Bi-FaSSIF			Buffer	
**t [min]**	**0**	**30**	**80**	**0**	30	65	0	30
k_disper_ [min^−1^]	**0.045**	0.045	0.045	**0.045**	0.045	0.045	**0.045**	0.045
k_dis1_ [min^−1^]		**0.009**	0.009		**0.009**	0.009		**0.009**
k_prec1_ [min^−1^]		2.0	2.0		2.0	2.0		2.0
k_dis2_ [min^−1^]			**0.04**			**0.05**		
k_prec2_ [min^−1^]			0.6			0.70		
k_part_ [min^−1^]		0.015	0.45		0.015	0.55		0.023
sat		3% (12 µg/mL)	3% (12 µg/mL)		5% (20 µg/mL)	5% (20 µg/mL)		2–5% (10 µg/mL)
max			35%			32%		

## Supplementary Materials

The following are available online at https://www.mdpi.com/1999-4923/12/3/237/s1. Figure S1: Light scattering measurement as an indicator of the dynamic behaviour of small nano-droplets.
