# Supplementary material for: A Rational Design of a Biphasic Dissolution Setup—Modelling of Biorelevant Kinetics for a Ritonavir Hot-Melt Extruded Amorphous Solid Dispersion
Source: Pharmaceutics. 2020 Mar 6;12(3):237. doi: 10.3390/pharmaceutics12030237 (PMC7150762; doi:10.3390/pharmaceutics12030237)
Supplement: Supplementary file 1 [file pharmaceutics-12-00237-s001.pdf]

Supplementary Data

Light Scattering Measurement as an Indicator of the Dynamic Behaviour of Small Nano-Droplets.

Changing nano-droplets properties could be also deduced from scattering (Figure 1) as undissolved particles cause light scattering, which results in turbidity. In the UV-Vis spectrum, this effect was evidenced by an exponential trend of the baseline of the UV-spectrum, which increases with smaller wavelengths. Hence, the scattering extent related turbidity throughout the experiment correlated with generation, growth or dissolution of particles e.g., nano-droplets.

As the aqueous phase was constantly pumped through a 1 μm cannula full-flow filter, particles of > 1 μm could not account for the scattering in the flow through cuvette (Figure 1). Starting at wavelength of > 300 nm, the UV-Vis spectrum results solely from light scattering as no absorbance of ritonavir is present. The extent of scattering was measured as turbidity values between wavelengths of 400 and 1000 nm (in 100 nm steps) during the entire experimental period. Consequently, when turbidity increased or decreased, it had to be related to LLPS, dissolution of nano-droplets, or a change in particle size.

The scattering profile of the plain buffer medium showed turbidity only during the gastric phase (Figure 1B). Later, the turbidity of the buffer medium (M3) rapidly decreased and remained at a low level, which indicated a fast-increasing particle size, because large undissolved ritonavir particles could not pass the filter anymore (Figure 1B). As a result, the partitioning of ritonavir from the plain buffer medium in the organic phase was slow.

In contrast, the scattering profile of the Bi-FaSSIF-V2 medium was highly dynamic (Figure 1A). The generally higher level of scattering in the Bi-FaSSIF-V2 was additionally affected by the Bi-FaSSIF-V2 ingredients. After shifting the pH to 5.5, scattering rapidly increased because LLPS of ritonavir into nano-droplets likely occurred. The decrease of scattering in Bi-FaSSIF-V2 started intensively before the partitioning rate increased. The decrease in turbidity resulted on the one hand from the fact that the large nano-droplets no longer passed the filter and on the other hand from the dissolution and Ostwald ripening of the small nano-droplets. The subsequent increased partitioning rate of ritonavir could be explained by an increased dissolution rate of shrunken small nano-droplets, which is represented in the kinetic model with the transformation of large nano-droplet (M_{lND}) into small nano-droplets (M_{sND}).

In summary, monitoring of scattering data is an easy and effective method to qualitatively measure the extent and kinetics of LLPS, particle size modification, and re-dissolution of nano-droplets, which at the same time confirm the validity of our kinetic model.

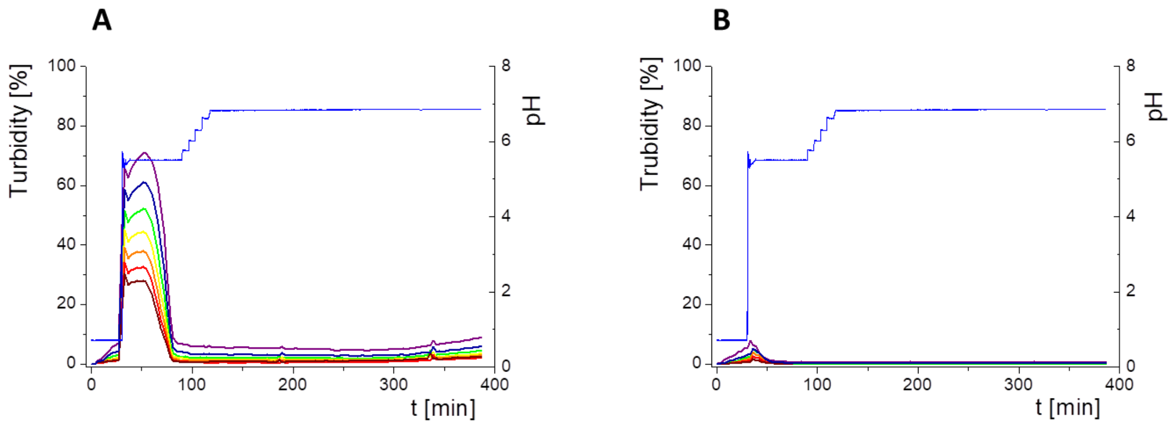

Figure S1: Turbidity in % $(\mathbf{1} - \frac{\textbf{\textit{light intensity of sample}}}{\textbf{\textit{light intensity of blank}}})$ in the course of the experiment caused by light scattering of particles smaller than 1 µm measured at various wavelengths; (**A**) is the scattering profile in Bi-FaSSIF-V2 medium; (**B**) represents the scattering profile in the surfactant free buffer medium. The colours of each turbidity profile resemble the colour in the electromagnetic spectrum: Purple 400 nm, blue 500 nm, green 600 nm, yellow 700 nm, orange 800 nm, red 900 nm, dark-red 1000 nm.
